# FunSet: an open-source software and web server for performing and displaying Gene Ontology enrichment analysis

**DOI:** 10.1186/s12859-019-2960-9

**Published:** 2019-06-27

**Authors:** Matthew L. Hale, Ishwor Thapa, Dario Ghersi

**Affiliations:** School of Interdisciplinary Informatics, College of Information Science & Technology, University of Nebraska at Omaha, 1110 S 67TH, Omaha, 68182 NE USA

**Keywords:** Gene Ontology, Web Tools, Functional Enrichment

## Abstract

**Background:**

Gene Ontology enrichment analysis provides an effective way to extract meaningful information from complex biological datasets. By identifying terms that are significantly overrepresented in a gene set, researchers can uncover biological features shared by genes. In addition to extracting enriched terms, it is also important to visualize the results in a way that is conducive to biological interpretation.

**Results:**

Here we present FunSet, a new web server to perform and visualize enrichment analysis. The web server identifies Gene Ontology terms that are statistically overrepresented in a target set with respect to a background set. The enriched terms are displayed in a 2D plot that captures the semantic similarity between terms, with the option to cluster terms via spectral clustering and identify a representative term for each cluster. FunSet can be used interactively or programmatically, and allows users to download the enrichment results both in tabular form and in graphical form as SVG files or in data format as JSON or csv. To enhance reproducibility of the analyses, users have access to historical data for the ontology and the annotations. The source code for the standalone program and the web server are made available with an open-source license.

## Background

Gene Ontology (GO) [1] enrichment analysis represents an effective way to tame the complexity of biological datasets and to facilitate their interpretation. The underlying idea is to identify sets of GO terms that are statistically overrepresented in a gene set of interest (e.g., a set of differentially expressed genes in an RNA-seq experiment or a set of genes associated with a trait in a genome-wide association study).

In order for GO enrichment analysis to be of value to biologists and biomedical researchers, it is important to have access to tools that allow users to perform the analysis and effectively display and interact with the results. Reproducibility of the results is another critical requirement in GO enrichment analysis, as it has been shown that the GO controlled vocabulary is significantly changing over time, in ways that affect the results of the analyses [2].

Here we present FunSet, a new web server for performing GO enrichment analysis on gene sets and interactively displaying the results. The tool allows users to optionally cluster the results using a spectral clustering algorithm and to extract representative terms for each cluster. In addition to these features, FunSet enables users to choose previous versions of the GO vocabulary and corresponding annotations. The goal of this “time machine” feature is to foster reproducibility of GO analyses, which – as mentioned above – have been shown to be sensitive to the version of the ontology and annotation used [2]. A comparison of FunSet with existing GO enrichment analysis tools is shown in Table 1.

**Table 1:** Gene Ontology Enrichment Analysis tools

Tools	Standalone	Open Source	Hist. data	Enrichment calc.	Background Set	Clusters	Interactive Plots
DAVID [3]	Windows XP/2K	No	Limited	Yes	Yes	Yes	No
REVIGO [4]	No	No	No	No	NA	Yes	Yes
WebGestalt [5]	No	No	No	Yes	Yes	No	No
Babelomics 5 [6]	No	No	No	Yes	Yes	No	No
PantherDB [7]	No	No	No	Yes	Yes	No	Yes
GORILLA [8]	No	No	No	Yes	Yes	No	No
FunSet	Yes	Yes	Yes	Yes	Yes	Yes	Yes

The table compares the main features of GO enrichment web servers, including: (1) availability of a standalone tool; (2) availability of the web server source code as open source software; (3) option to choose historical GO data; (4) enrichment analysis calculations; (5) option to define a custom background set; (4) clustering of the terms; (5) interactive visualization

FunSet can be used programmatically with an API or from the command line. The source code for the entire pipeline (including the web server) is made available with an open source license.

In summary, the contribution of FunSet are: (1) “time machine” feature that allows users to use GO historical data for reproducibility; (2) interactive visualization with clustering of terms and automatic identification of an optimal number of clusters and representative terms; (3) availability of the source code for both the command line programs and the web interface, enabling users to extend the pipeline or incorporate it into other existing pipelines.

A description of the implementation follows.

## Methodology and implementation

In order to perform GO enrichment analysis, FunSet requires users to specify: (1) an organism; (2) a target set; (3) a background set (optional); (4) an FDR threshold for multiple hypothesis correction; (5) the GO namespace (one of “biological process”, “molecular function”, or “cellular component”); and (6) an ontology/annotation version.

### Input data

#### Organisms

FunSet currently supports the following organisms, which can be selected from a pull-down menu: *Homo sapiens* (human), *Gallus gallus* (chicken), *Bos taurus* (cow), *Canis familiaris* (dog), *Mus musculus* (mouse), *Rattus norvegicus* (rat), *Caenorhabditis elegans* (nematode), *Arabidopsis thaliana* (thale cress), *Drosophila melanogaster* (fruit fly), *Saccharomyces cerevisiae* (budding yeast), and *Danio rerio* (zebrafish).

#### Gene sets

Enrichment analysis requires a target set (i.e., genes with a property of interest) and a background set. The user is required to enter the target set either as a comma-separated list in a text box or by uploading a text file. Optionally, the user can also upload a background gene set. Otherwise, by default FunSet will select as background all annotated genes for the chosen organism. The accepted format for specifying genes is HGNC symbols [9] for human, VGNC symbols [9] for cow and dog, and MOD (model organism databases) symbols [10] for model organisms.

#### FDR threshold

FunSet handles multiple comparisons using the Benjamini-Hochberg procedure [11]. The user has the option to enter a specific False Discovery Rate (FDR) threshold to filter the results; otherwise, FunSet uses the default threshold of 0.05.

#### Ontology version

In order to facilitate the reproducibility of published results, FunSet allows the user to select historical versions of the GO controlled vocabulary and organism annotations.

### Enrichment analysis

The per-term enrichment analysis is performed using the hypergeometric distribution, which models sampling without replacement:

1 $$ P(X \ge k) = \sum_{x=k}^{min(K, n)}\frac{{K \choose x} {N-K \choose n-x}}{{N \choose n}}  $$

where *P*(*X*≥*k*) is the probability of observing at least *k* genes with a given GO term, *N* is the total number of genes in the background set, *K* is the total number of genes annotated with the given term, *n* is the total number of genes in the target set, and *x* is the total number of genes in the target set annotated with the given term.

### Clustering of terms

FunSet can also perform clustering of significantly enriched terms, in order to identify semantically similar groups of terms. The first step involves computing the semantic similarity between all pairs of enriched terms using the Aggregate Information Content (AIC) [12], an index that takes into consideration the information content of all ancestral terms of a GO term in the graph. The AIC index has been shown to perform better than other widely used measures of semantic similarity [12].

In the command line version of the program the user can also choose to use the Lin Index [13]. The Lin Index ranges from 0 (semantically unrelated terms) to 1 (semantically identical terms), and is computed as follows:

2 $$ \text{Lin}(t_{1}, t_{2}) = \frac{2\times IC(c)} {(IC(t_{1}) + IC(t_{2}))}  $$

where *c*∈*S* and *S* is the set of Lowest Common Ancestors (LCAs) of the two terms with the maximum Information Content (IC). The IC of a term *t*_*i*_ is calculated as:

3 $$ IC(t_{i}) = -\log(p_{t_{i}})  $$

where $p_{t_{i}}$ is the probability of the term *t*_*i*_, calculated as the number of genes annotated with *t*_*i*_ or with an ancestor term of *t*_*i*_ divided by the total number of annotated genes.

A matrix containing the pairwise semantic similarity between all enriched terms is then created and used to cluster the terms with the spectral cluster algorithm implemented in the scikit-learn [14] Python package, using default parameters and the desired number of clusters provided by the user. If the user does not specify a desired number of clusters, FunSet will estimate an optimal number using the eigengap strategy proposed by von Luxburg [15].

Finally, FunSet selects the medoids of each cluster, i.e., the terms with the largest average semantic similarity with respect to all terms in the cluster, as cluster representatives.

### JavaScript Object Notation Application Programming Interface (JSONAPI)

FunSet is, at its core, a RESTful web service that meets the JSONAPI standard [16]. JSONAPI is a prescriptive format and protocol that sits on top of HTTP and promotes well-defined multi-platform interoperability by eliminating the need for ad-hoc code to be defined on a per-application basis. FunSet uses JSONAPI as a means to execute an analysis pipeline, translate the analysis data into a web-serialized format, and to pipe it to a frontend web visualization interface, described below in a later section. In addition, the FunSet web service also exposes its underlying capabilities publically, allowing users to programmatically invoke the enrichment and clustering process and receive results as raw JSON.

#### API endpoints

The FunSet API is organized around a set of *API endpoints* that can be invoked programmatically using a REST client, such as POSTMAN [17], using any http command line tool, such as CURL [18], or via the web using the visualization client application. The endpoints it provides are documented below. Each endpoint accepts HTTP GET and/or POST requests. Endpoint documentation below uses the following notational syntax:


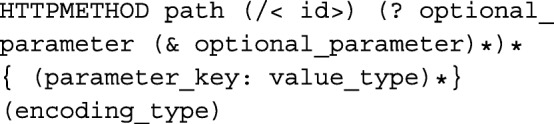
 where the parenthetical, (), denotes a pattern that occurs 0-1 times, the wildcard parenthetical notation, ()∗, indicates the pattern occurs 0 or more times, *httpmethod* is either GET or POST, *path* is a relative url from root (e.g. *funset*.*uno*/*path*) that idenfies the corresponding API endpoint, <*id*> is the unique id of the object (where applicable), an *optional*_*parameter* is a url-encoded parameter the endpoint optionally accepts, (*parameter*_*key*:*value*_*type*)∗ is a list of required parameters (e.g. POST parameters) that, where applicable, are encoded following the *encoding*_*type*. All API endpoints are accessible without login to faciliate open-access.


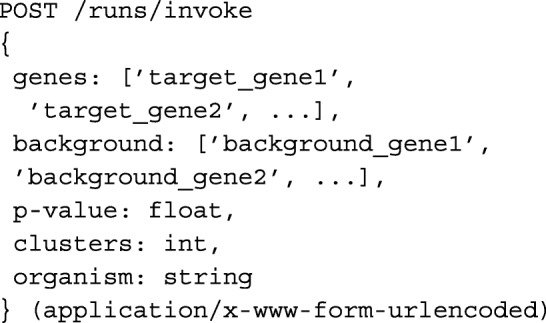


The *runs*/*invoke* method is the primary endpoint on the API and facilitates the creation of a new **run** object, following the schema defined below, in a JSON format. Broadly speaking, a **run** is an object that encapsulates the results of an instantiation of the enrichment and spectral clustering algorithm. In this way, **run** contains the results of execution as a set of enriched terms, each of which is represented as an **enrichment** object, following the schema below. The *runs*/*invoke* endpoint will produce well defined output enrichments when the POST parameters take on any of the following values:

- all gene strings in the genes list are valid GO gene ids;
- all gene strings in the background list are valid GO gene ids;
- the p-value, representing the false detection rate to use for the run is a float between 0 and 1;
- the clusters parameter is an integer from 1 to the total number of target genes supplied, representing the desired number of clusters to use in the spectral clustering algorithm, or -1 for automatic detection of the optimal number; and
- the organism parameter is one of the following 3-letter codes: [’hsa’, ’gga’, ’bta’, ’cfa’, ’mmu’, ’rno’, ’cel’, ’ath’, ’dme’, ’sce’, ’eco’, or ’dre’]

To retrieve the data for each of the enriched terms, one should make an additional request to the GET /enrichments endpoint defined below, for each enriched term id listed in the *run*.*enrichments* field.


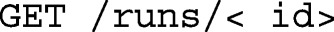
 returns a previously completed **run** object specificed by the <*id*> or a 404 Not Found error, if the <*id*> does not point to a valid **run** object.


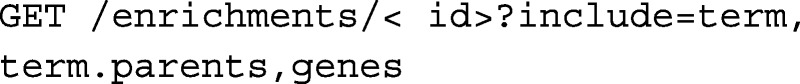
 returns an enrichment term’s data, whose primary key is <*id*>, corresponding to the **enrichment** schema below or a 404 if the term specified by the does not exist. If passed the *include* parameter with *term*, *term*.*parents*, and/or *genes*, the method will also fetch and return all related term and gene fields, see **term** and **gene** schemas, respectively, below.


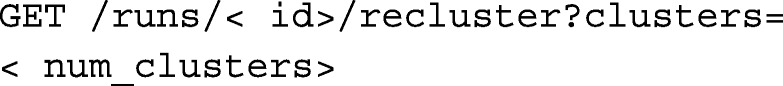
 re-runs the spectral clustering algorithm for an existing run specified by <*id*>, grouping terms into a number of clusters equal to *num*_*clusters* as specified by the url encoded parameter *clusters*, where *num*_*clusters* must be a number between 1 and the total number of terms in the background set. This method returns a **run** object with the same structure as /runs/invoke, or returns 404 Not Found if the run specified by the <*id*> is not an extant valid run.


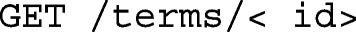
 returns GO term data, following the **term** schema below, for the term matching the <*id*>, or a 404 Not Found error if the term does not exist.


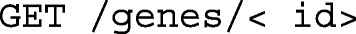
 returns gene data, matching the **gene** schema below, for the gene specified by the <*id*>, or a 404 Not Found error if the id is invalid.

**Gene**

- id (int)
- name (string)

Table 2 shows Funset’s API data schema.

**Table 2:** API Data Schema

Run	Enrichment	Term
**id** (int)	**id** (int)	**id** (int)
**created** (date)	**created** (date)	**name** (string)
**ip** (string, requestor’s IP)	**term** (id (int), defining a ForeignKey to **Term**)	**termid** (string, official GO id)
	**pvalue** (float - detection rate in sample)	
**enrichments** (list of id (int), defining a one-to-many relationship to **Enrichment**)	**level** (float - enrichment level in sample)	**namespace** (string)
	**semanticdissimilarityx** (float - x position of term in graph scaled to [0-1])	**description** (string)
	**semanticdissimilarityy** (float - y position of term in graph scaled to [0-1])	**synonym** (string)
	**cluster** (int - the cluster to which the enriched term is assigned)	**parents** (list of id (int), defining a many-to-many relationship to **Term**)
	**medoid** (boolean - true if this term is the medoid of its cluster)	
	**genes** (list of id (int), defining a one-to-many relationship to **Gene** that represents all genes enriched in the sample)	

The boldface items represent the data field names (i.e., the fields in the schema)

### Visualization Techniques

To visualize the results of the GO enrichment analysis, we built a client-side front-end as a web application using Ember.js [19, 20] and D3.js [21]. The web application allows users to specify a target gene set, a background gene set, p-value and an ontology, namespace, and organism to be used for enrichment analysis. Given the user selections, the web application invokes the *runs/invoke* API described above, mapping the user selections in the interface to the input parameters as specified. The JSON results returned by the API are then rendered into an SVG visualization. The FunSet visualization represents terms in a 2D coordinate space, where terms are positioned using Multidimensional Scaling (MDS) on the distance matrix obtained from the pairwise AIC semantic similarity index described before. A term’s x,y coordinate location in the svg is characterized by the following formula.

4 $$ (x,y) = ({svg}_{w}*{sc}_{x}, {svg}_{h}*{sc}_{y})  $$

where *svg*_*w*_ and *svg*_*h*_ are, respectively, the pixel width and height of the svg as it fits in the user’s browser and *sc*_*x*_ and *sc*_*y*_ are, respectively, the spectral clustering *x* and *y* results, ranging from 0 to 1. In effect, this scales the SVG to the user’s browser size, while maintaining the original, location significant, aspect ratio. Node size in the visualization graph is scaled according to the enrichment size effect produced by the enrichment analysis. The enrichment size for a term is calculated as the number of genes associated with a term in the target set divided by the expected number of genes.

After setting initial term locations to be the scaled clustering location, FunSet’s visualization interface then applies a velocity Verlet using D3’s force library [22] to each term to distribute terms away from one another, uniformly, within the SVG space. This technique is used to mitigate scenarios where terms are tightly stacked within a cluster - making visual interpretation difficult. The Verlet numerical integrator used in FunSet simulates physical motion of terms in the SVG by applying a constant acceleration *a* over a time interval *Δt* to the term’s velocity, changing its (x,y) position at each time step. With velocity initially set to 0, this accelerates terms in the graph by adding *a* to the term’s velocity at each time step. To disperse terms, without disrupting the underlying cluster structure, we apply a uniform repulsive force to each term that simulates magnetic repulsion. At the same time, a link-force is applied for terms with parent/child relationships in the data. Finally, a decay function simulating physical friction stabilizes the graph and allows it to reach a steady state. The entire physics simulation is compute optimized to perform well even for large networks of enriched terms.

FunSet auto-expands the cluster and term panels and then jumps to the enriched term’s reference material on the right-hand side when a user clicks a node to inspect it further. GO terms are linked to Amigo [23] so that users can jump directly to the external GO term reference page.

## Results

### Running the GO enrichment analysis and visualizing the results

**Fig. 1:**
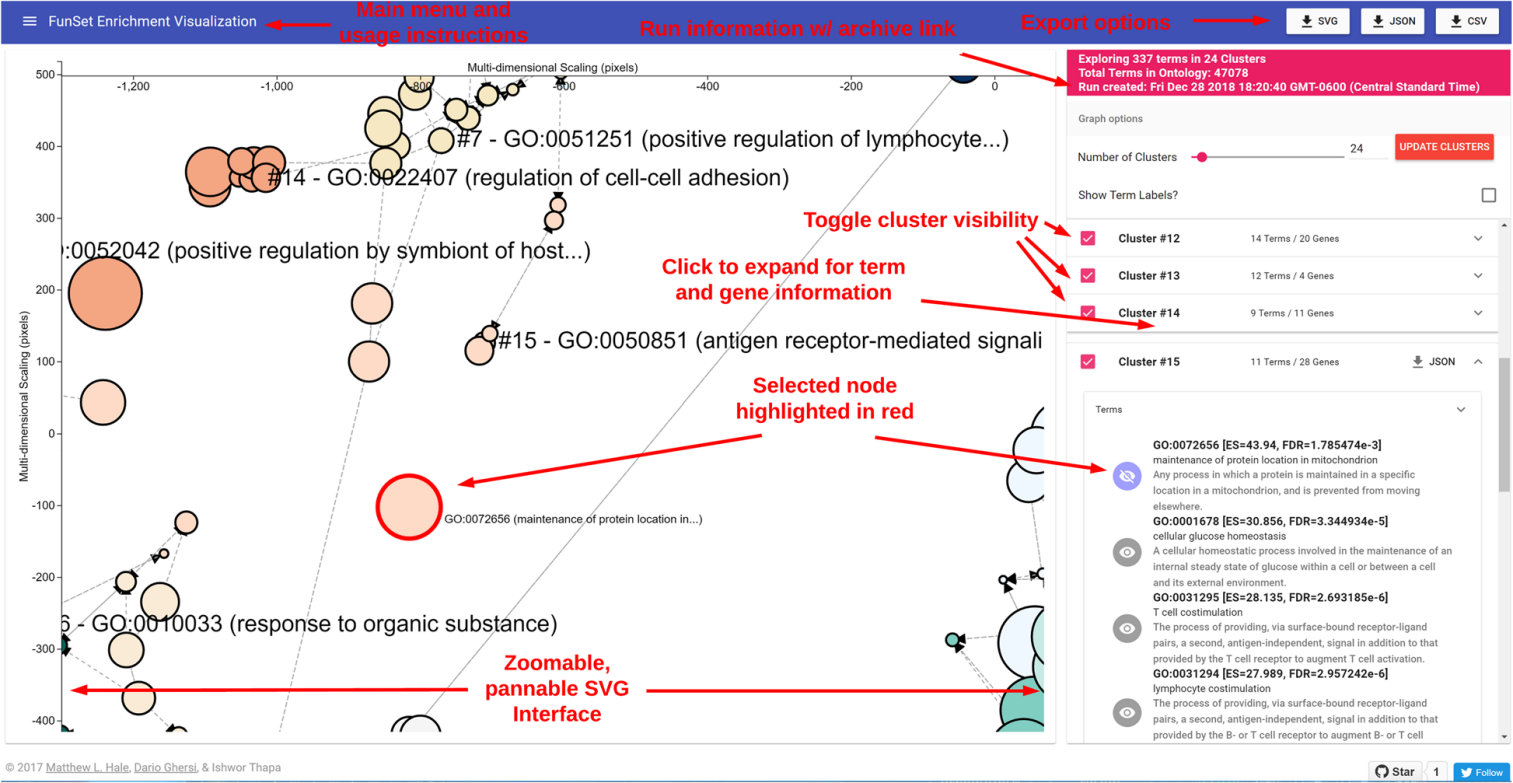
FunSet’s User Interface. The figure shows the results of GO enrichment analysis, with the network view of the terms on the left and the toggeable clusters/terms panel on the right

Figure 1 shows an overview of the FunSet visualization user interface. The SVG space with the clusters of terms is shown on the left. This area is pannable and zoomable by left clicking and dragging or using the mouse wheel, respectively. The right hand side of the interface shows information about the computed enrichment analysis, including the time when the run was created, which is clickable to copy a permanent link that the user can use to return to this run data, the total number of terms within the ontology data used, the total terms that were found to be enriched, and then the set of clusters the enriched terms fell into. The interface allows the user to change the number of desired clusters using the right hand slider. The interface also allows the user to show or hide clusters by toggling the cluster visibility buttons.

Clicking a cluster will expand it to show the enriched terms with their associated data such as the false discovery rate *FDR* and the enrichment size *ES*. The term panel allows users to click a particular term to highlight it, in red, in the SVG graph. Clicking a term in this panel will also display the term’s description. A second panel (not shown in the figure) shows the specific genes contributing to the enrichment for each cluster.

The visualization UI also allows the user to export the results of a run as an SVG, as JSON, and as a CSV. Both the JSON and CSV data structures follow a hierarchical format consistent with the API description. The interface also allows a user to export JSON data regarding a particular cluster. The interface also allows users to click nodes in the graph to expand their term information within a cluster.

### Case study: comparing enrichment analysis results across time

**Fig. 2:**
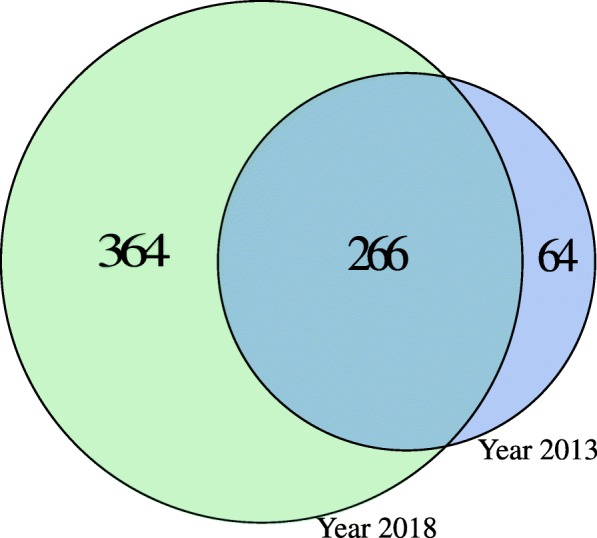
Comparison of GO enrichment analysis performed at different time points. The Venn diagram shows the overlap between significantly enriched terms (FDR <0.05)

A study by Wadi et al. showed that outdated enrichment tools could only recover 26% of biological processes and pathways identified with more up-to-date resources [2]. As a proof-of-principle, we used FunSet to perform GO enrichment analysis in the “biological process” namespace on a list of predicted cancer “driver” genes [24] using 2013 and 2018 GO vocabulary and annotations, respectively. The results show a substantial difference in the number of enriched terms, with 364 gained terms with respect to the 2013 version, and 64 “lost” terms (Fig. 2).

**Fig. 3:**
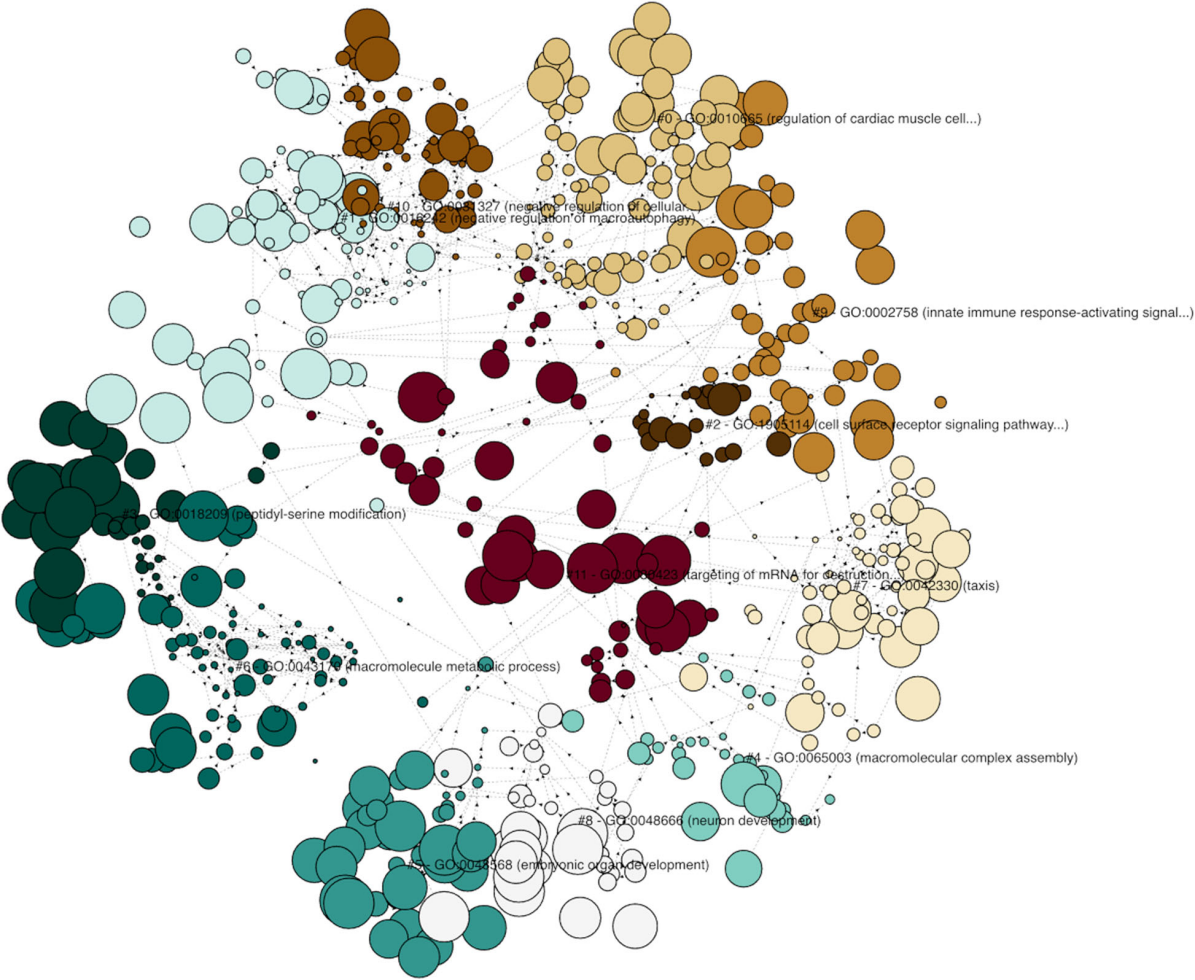
Clustering of enriched tems. The list of predicted cancer driver genes in [24] yields 630 enriched GO terms in the biological process namespace using 2018 GO data. Funset automatically identified 12 representative clusters using the eigengap approach [15]

We used the same list of genes to highlight how clustering can help to summarize long list of enriched terms. As shown in Fig. 3, FunSet automatically identified twelve clusters of terms, and returned the representative (medoid) term for each cluster. The representative terms are shown in Table 3.

**Table 3:** Representative terms (medoid terms) in the biological process namespace automatically identified by FunSet for the gene list reported in [24]

ClusterID	GO ID	GO Term	# of Terms	# of Genes
0	GO:0010665	Regulation of cardiac muscle cell apoptotic process	80	31
1	GO:0016242	Negative regulation of macroautophagy	76	25
2	GO:1905114	Cell surface receptor signal. pathway involved in cell-cell signal.	20	19
3	GO:0018209	Peptidyl-serine modification	35	22
4	GO:0065003	Macromolecular complex assembly	33	27
5	GO:0048568	Embryonic organ development	43	23
6	GO:0043170	Macromolecule metabolic process	75	34
7	GO:0042330	Taxis	66	33
8	GO:0048666	Neuron development	40	19
9	GO:0002758	Innate immune response-activating signal transduction	48	31
10	GO:0031327	Negative regulation of cellular biosynthetic process	52	21
11	GO:0030423	Targeting of mRNA for destruction involved in RNA interf.	62	34

The full list of enriched terms contains 630 terms using the 2018 GO release

## Discussion

Enrichment analysis is a widely used bioinformatics approach that enables experimental and computational investigators to extract meaningful information from long lists of genes. Here we introduced FunSet, a new web server for performing and visualizing GO enrichment analysis interactively through a web server, programmatically via an API, or from the command line. We also discussed a case study that illustrates the impact of time (and therefore different versions of the GO vocabulary and annotations) on the results of otherwise identical enrichment analyses. This points to the importance of using time-stamped versions of the GO vocabulary and corresponding annotations when attempting to reproduce computational analyses. To the best of our knowledge, this is the first time that a comprehensive tool for GO enrichment analysis and visualization allows users to use historical GO data. The case study also illustrates the use of clustering to identify meaningful groups of terms that can be summarized with one representative term per cluster, automatically chosen by FunSet. We note that while FunSet can determine an optimal number of clusters with the eigengap procedure [15], users still have the option (and are encouraged) to explore with different number of clusters, to identify groups of terms that match their biological intuition at the desired granularity level.

## Conclusions

We have introduced a novel tool named FunSet to perform and visualize GO enrichment analysis. By having access to the full documented source code of the pipeline, users can deploy FunSet on a private cloud for increased computational performance, and potentially customize it using other controlled vocabularies. Further, the availability of a documented, open-source standalone program allows users to incorporate FunSet into other bioinformatics pipelines or extend its features.

## Availability and requirements

**Project name**: FunSet

**Project home page**: http://funset.uno

**Operating system(s)**: Platform independent (web server); Linux, Mac OS X (command-line software)

**Programming language**: Python, C++, JavaScript

**Other requirements**: none

**License**: GPL-3

**Any restrictions to use by non-academics:** none

## Data Availability

The web server is available at http://funset.uno. Source code for the web server and the standalone programs is available at https://github.com/mlhale/funset-enrichment-visualization. The gene lists used in the examples can be found in the examples within the source code archive.

## Abbreviations

FDR: False discovery rate
GO: Gene ontology
IC: Information content
LCA: Lowest common ancestor

## References

[1] Ashburner M, Ball CA, Blake JA, Botstein D, Butler H, Cherry JM, Davis AP, Dolinski K, Dwight SS, Eppig JT, Harris MA, Hill DP, Issel-Tarver L, Kasarskis A, Lewis S, Matese JC, Richardson JE, Ringwald M, Rubin GM, Sherlock G (2000). Gene ontology: tool for the unification of biology. The Gene Ontology Consortium. Nat Genet.

[2] Wadi L, Meyer M, Weiser J, Stein LD, Reimand J (2016). Impact of outdated gene annotations on pathway enrichment analysis. Nat Methods.

[3] Huang DW, Sherman BT, Lempicki RA (2009). Systematic and integrative analysis of large gene lists using DAVID bioinformatics resources. Nat Protoc.

[4] Supek Fran, Bošnjak Matko, Škunca Nives, Šmuc Tomislav (2011). REVIGO Summarizes and Visualizes Long Lists of Gene Ontology Terms. PLoS ONE.

[5] Wang J, Vasaikar S, Shi Z, Greer M, Zhang B (2017). WebGestalt 2017: A more comprehensive, powerful, flexible and interactive gene set enrichment analysis toolkit. Nucleic Acids Res.

[6] Alonso R, Salavert F, Garcia-Garcia F, Carbonell-Caballero J, Bleda M, Garcia-Alonso L, Sanchis-Juan A, Perez-Gil D, Marin-Garcia P, Sanchez R, Cubuk C, Hidalgo MR, Amadoz A, Hernansaiz-Ballesteros RD, Alemán A, Tarraga J, Montaner D, Medina I, Dopazo J (2015). Babelomics 5.0: Functional interpretation for new generations of genomic data. Nucleic Acids Res.

[7] Mi H, Huang X, Muruganujan A, Tang H, Mills C, Kang D, Thomas PD (2017). PANTHER version 11: Expanded annotation data from Gene Ontology and Reactome pathways, and data analysis tool enhancements. Nucleic Acids Res.

[8] Eden E, Navon R, Steinfeld I, Lipson D, Yakhini Z. GOrilla: A tool for discovery and visualization of enriched GO terms in ranked gene lists. BMC Bioinformatics. 2009; 10. 10.1186/1471-2105-10-48. 10.1186/1471-2105-10-48 PMC2644678 19192299

[9] Yates Bethan, Braschi Bryony, Gray Kristian A., Seal Ruth L., Tweedie Susan, Bruford Elspeth A. (2016). Genenames.org: the HGNC and VGNC resources in 2017. Nucleic Acids Research.

[10] Oliver SG, Lock A, Harris MA, Nurse P, Wood V. Model organism databases: Essential resources that need the support of both funders and users. BMC Biol. 2016. 10.1186/s12915-016-0276-z. 10.1186/s12915-016-0276-z PMC4918006 27334346

[11] Benjamini Y, Hochberg Y (1995). Controlling the False Discovery Rate: A Practical and Powerful Approach to Multiple Testing. J R Stat Soc Ser B (Methodol).

[12] Song Xuebo, Li Lin, Srimani Pradip K., Yu Philip S., Wang James Z. (2014). Measure the Semantic Similarity of GO Terms Using Aggregate Information Content. IEEE/ACM Transactions on Computational Biology and Bioinformatics.

[13] Lin D. An Information-Theoretic Definition of Similarity. Proc ICML. 1998. https://doi.org/10.1.1.55.1832.

[14] Pedregosa F, Varoquaux G, Gramfort A, Michel V, Thirion B, Grisel O, Blondel M, Prettenhofer P, Weiss R, Dubourg V, Vanderplas J, Passos A, Cournapeau D, Brucher M, Perrot M, Duchesnay E (2011). Scikit-learn: Machine learning in Python. J Mach Learn Res.

[15] von Luxburg Ulrike (2007). A tutorial on spectral clustering. Statistics and Computing.

[16] JSON:API. JSON API. https://jsonapi.org/. Accessed 1 Mar 2019.

[17] Wagner J. Review: Postman Client Makes RESTful API Exploration a Breeze: Program; 2014. https://www.programmableweb.com/news/review-postman-client-makesrestful-api-exploration-breeze/brief/2014/01/27. Accessed 1 Mar 2019.

[18] Everything curl. CURLOPT_READFUNCTION. https://curl.haxx.se/. Accessed 1 Mar 2019.

[19] Ember.js. Ember JS. https://emberjs.com/. Accessed 1 Mar 2019.

[20] Skeie JH (2014). Ember. js in Action.

[21] Bostock M (2012). D3. js. Data Driven Doc.

[22] D, 3 Force library. D3 Documentation. https://github.com/d3/d3-force. Accessed 1 Mar 2019.

[23] Carbon Seth, Ireland Amelia, Mungall Christopher J., Shu ShengQiang, Marshall Brad, Lewis Suzanna (2008). AmiGO: online access to ontology and annotation data. Bioinformatics.

[24] Ghersi Dario, Singh Mona (2013). Interaction-based discovery of functionally important genes in cancers. Nucleic Acids Research.

